# Persistence of hepatitis C virus in peripheral blood mononuclear cells of patients who achieved sustained virological response following treatment with direct-acting antivirals is associated with a distinct pre-existing immune exhaustion status

**DOI:** 10.1038/s41598-025-05084-z

**Published:** 2025-06-06

**Authors:** Sylwia Osuch, Marta Kazek, Paulina Emmel, Hanna Berak, Marek Radkowski, Kamila Cortés-Fendorf

**Affiliations:** 1 Department of Immunopathology of Infectious and Parasitic Diseases, Medical University of Warsaw, 3C Pawińskiego Street, 02-106 Warsaw, Poland; 2 Laboratory of Genetics, University Clinical Center of the Medical University of Warsaw, Warsaw, Poland; 3 Outpatient Clinic, Warsaw Hospital for Infectious Diseases, Warsaw, Poland

**Keywords:** Occult HCV infection, Sustained virologic response (SVR), Direct-acting antivirals (DAA), Immune exhaustion, Genetics, Immunology, Microbiology, Molecular biology

## Abstract

Hepatitis C virus (HCV) is a primary hepatotropic pathogen responsible for acute and chronic hepatitis C, however, it can also cause “occult” infection (OCI), defined as the presence of the virus’ genetic material in hepatocytes and/or peripheral blood cells, but not in plasma/serum. Assessment of the sustained virologic response (SVR) after treatment with direct-acting antivirals (DAA) is based exclusively on HCV-RNA testing in plasma/serum, which may preclude the diagnosis of post-treatment OCI. Possible clinical consequences of OCI were described previously, but its occurrence after DAA-based antiviral treatment programs and determinants of the virus persistence are not fully elucidated. The aim of this study was to assess the incidence of post-treatment OCI after successful DAA-based treatment and to identify clinical and immunological factors associated with this phenomenon. In 97 patients treated with DAA, HCV-RNA was tested by RT-PCR in peripheral blood mononuclear cells (PBMC) at baseline (i.e., before the onset of treatment) and at the time of SVR assessment. Before treatment, HCV-RNA was detectable in all patients’ PBMC. All subjects responded to therapy according to the clinical criteria, but 9 (9.3%) patients revealed the HCV-RNA in PBMC at SVR. In most of these cases, post-DAA OCI was related to switch of the dominant infecting genotype. Post-treatment OCI was characterized by significantly lower pre-treatment HCV viral load and lower expression of Tim-3 (T-cell immunoglobulin and mucin domain-containing protein 3) on CD8^+^ T-cells. Our results imply that post-treatment OCI may be related to lower pretreatment viral load as well as distinct pre-existing immune exhaustion status.

**Supplementary Information:**

The online version contains supplementary material available at 10.1038/s41598-025-05084-z.

## Introduction

Hepatitis C virus (HCV) is a cause of chronic hepatitis C (CHC), which may lead to liver cirrhosis and hepatocellular carcinoma (HCC). Although hepatocytes are the primary site of HCV infection, CHC is increasingly recognized as a systemic disease, associated with a wide spectrum of accompanying symptoms^1,2^. Secondary tropism and extrahepatic replication have been demonstrated in PBMCs, macrophages, dendritic cells, bone marrow and central nervous system^3–7^.

The “occult” HCV infection (OCI) is defined as the presence of the virus’ genetic material in hepatocytes and/or peripheral blood cells, and not in plasma/serum^8,9^. Viral replication usually occurs at a low level^10^, and the presence of anti-HCV antibodies can be either positive or negative^8,9^.

OCI is prevalently observed in patients in whom the infection has resolved in the acute phase (up to 70% of cases) or who were treated with previous interferon-α-based therapies^8,11,12^. Studies conducted on the general population revealed OCI in 3% of apparently healthy subjects and in 2% of blood donors^13^. OCI has been described as transient, recurrent, or long-term and patients rarely manifest clinical symptoms and/or elevated liver enzymes^14^. Nevertheless, it may be also related to progressive inflammatory changes, liver fibrosis, and infection reactivation^15–24^. Although HCV from subjects with OCI was found to infect human lymphocytes from healthy donors^10^, there is not enough evidence data to whether OCI is transmissible.

The best approach for OCI diagnosis is based on the use of sensitive molecular methods detecting HCV RNA in liver tissue^10^. However, because of its invasive character, PBMC testing has been also employed, because of a good concordance between detection of HCV-RNA in PBMC and liver tissue, reaching 70%^13,16,25^.

Actual standard of CHC treatment is based on direct-acting antivirals (DAA) suppressing viral replication by inhibiting the activity of NS3 protease, NS5A protein or NS5B polymerase^21^. Treatment became shorter, less toxic and very effective, when compared to the previously used combination of interferon-α and ribavirin. Sustained virologic response (SVR), defined as a negative result of viral RNA testing in blood plasma/serum 12 or 24 weeks after the end of treatment, is achieved in > 90% of patients^26^. However, SVR is not always equal to the complete virus eradication and does not exclude the post-treatment OCI. Ongoing viral persistence following successful DAA treatment may lead to clinical progression of liver disease or relapse^22,27^. Thus, it is of importance to implement surveillance of patients who achieved SVR, as well as to investigate post-DAA OCI – including its possible determinants, which may lay both in the mechanisms of cell infection and the specific immune response of the host^16,28–31^. Similarly, since the risk of HCC development persists despite achieving SVR, OCI has been proposed as a potential risk factor. However, current evidence remains inconclusive and a larger, longitudinal studies are required to definitively assess whether persistent OCI may lead to hepatocarcinogenesis^32–38^.

Immune exhaustion is an immune cell dysfunction characteristic of chronic infections and cancer, originating from prolonged and high-level antigen exposure as well as inflammatory signals^39–42^. It was observed in chronic viral infections, such as HCV, HIV, HBV, adenovirus, polyomavirus, HTLV-1^43–47^ and affects both CD8^+^ and CD4^+^ T-cells, but was also observed in NK or B-cells^48,49^. In particular, CD8^+^ T cells undergo a progressive loss of effector functions, encompassing impaired proliferation and ability to secrete effector cytokines, loss of cytotoxicity, upregulated expression of inhibitory receptors (iRs) as well as dysregulated transcriptional program, contributing to impaired viral control^39,40,50^. Some key iRs are PD-1, Tim-3 and LAG-3. Progression of exhaustion is characterized by excessive, constitutive, simultaneous expression of multiple iRs as well as upregulation of IL-10^51,52^. In addition to membrane-bound molecules, soluble iRs (eg., sPD-1, sTim-3, sLAG-3) are detectable in plasma, mainly as a result of enzymatic cleavage, cells breakdown or alternative splicing^53–56^. Understanding the immunological landscape of exhaustion in patients with post-DAA OCI is therefore of clinical relevance.

The aim of this study was to assess the incidence of post-treatment OCI after successful DAA treatment and to analyze possible clinical and immunological factors associated with this phenomenon.

## Materials and methods

### The research material

PBMC isolated from the whole blood by density gradient centrifugation using the Lymphoprep reagent (Stemcell Technologies) of 97 CHC patients from the Warsaw Hospital for Infectious Diseases were collected on the day of treatment initiation and 24 weeks after completion of treatment with DAA, corresponding to the SVR24 assessment time point.

All subjects qualified for the study achieved SVR by HCV RNA testing using a PCR method of a sensitivity of 12 IU/mL (Abbott RealTime HCV Viral Load Assay). The following data were collected: age, sex, BMI, bilirubin levels, ALT activity, liver elastography (FibroScan), HCV genotype (Inno-LIPA HCV II, Innogenetics) and viral load before treatment (Abbott RealTime HCV Viral Load Assay). The characteristics of the study group are presented in Table 1.

**Table 1:** Characteristics of the study participants.

Age [median (range)]		57 (25–88)
BMI [kg/m^2^]		26.1 (15.0–46.7)
Sex [F/M]		61/36
Baseline bilirubin level [mg/dL] [median (range)]		13.1 (5.3–46.6)
ALT [IU/L] [median (range)]		61 (19–389)
Baseline viral load [IU/mL] [median (range)]		8.3 × 10^5^ (6.2 × 10^3^–1.1 × 10^7^)
CD4 per CD3^+^ cells [%]		65.9 (26.2–90.1)
CD8 per CD3^+^ cells [%]		22.8 (6.9–57.1)
HCV genotype	1a	2
	1b	95
Liver fibrosis stage	F0/1	56
	F2	27
	F3	14
Treatment scheme and duration	Harvoni (Ledipasvir + Sofosbuvir) (8/12 weeks)	69
	Viekirax + Exviera (Ombitasvir + Paritaprevir + Ritonavir + Dasabuvir) (8/12 weeks)	21
	Zepatier (Elbasvir + Grazoprevir) (12 weeks)	7
Previous unsuccessful treatment history	DAA	1
	interferon-α + ribavirin	23

The study was conducted in compliance with the Declaration of Helsinki and was approved by the bioethics committee of the Medical University of Warsaw (approval number: AKBE/43/2022). Written informed consent was obtained from all patients prior to the study initiation.

### Isolation of HCV RNA and degradation of contaminating DNA

RNA was isolated from PBMC samples containing 3 million cells by Chomczynski method using TRIzol reagent (Invitrogen) and resuspended in molecular biology grade water (Invitrogen). The DNA-free Kit (Ambion) was used in accordance with the manufacturer’s recommendations to degrade any contaminating DNA.

### Reverse transcription and amplification of the HCV 5’ UTR fragment by polymerase chain reaction (PCR)

DNA-free RNA was reversely transcribed using a M-MLV reverse transcriptase (Invitrogen) according to the manufacturer’s recommendations. A positive control template comprised synthetic 5’ UTR (5’ untranslated region) HCV RNA strands derived from a plasmid; negative control comprised a molecular biology grade water instead of a template.

A PCR reaction mixture was prepared using a forward (5′-TGRTGCACGGTCTACGAGACCTC-3′) and reverse (5′-RAYCACTCCCCTGTGAGGAAC-3′) reaction primers and FastStart Taq Polymerase Kit (Roche). One µl of cDNA was used as a reaction template. The PCR reaction comprised one cycle of initial denaturation at 94 °C for 5 min; 50 cycles, each consisting of denaturation for 1 min at 94 °C and primer annealing for 1 min at 58 °C, and one cycle of elongation for 7 min at 72 °C.

### Nested PCR and electrophoresis

The nested PCR was performed using forward (5′-ACTGTCTTCACGCAGAAAGCGTC-3′) and reverse (5′-CAAGCACCCTATCAGGCAGTACC-3′) primers. One µl of the PCR product was used as a reaction template. The PCR steps included: one cycle of initial denaturation at 94 °C for 5 min; 30 cycles, each consisting of denaturation for 1 min at 94 °C and primer annealing for 1 min at 58 °C, and final extension for 7 min at 72 °C. The PCR product (273 bp) was visualized by 2% agarose gel electrophoresis using the SYBR Safe DNA Gel Stain reagent (Invitrogen).

Based on the previous amplification of serial dilutions of synthetic HCV RNA strands, derived from a plasmid with cloned HCV 5’ UTR transcribed with T7 polymerase, the analytical sensitivity of the employed method was estimated to be ~ 10 genomic equivalents of the template^57,58^.

### 5’UTR amplicon sequencing

5′ UTR amplicons obtained from post-treatment PBMC samples along with their respective pre-treatment pairs were purified using Nucleospin PCR Clean-up and Gel Extraction Kit (Macherey–Nagel), quantified using the Qubit assay dsDNA HS kit (Thermo Fisher Scientific) and subjected to Sanger-based sequencing on ABI Prism Genetic Analyzer to detect dominant genotype strains.

### Next-generation sequencing (NGS) analysis

In cases in which a switch in the dominant genotype was detected in PBMC by Sanger-based sequencing, NGS of pre-treatment serum and PBMC samples was performed to search for minor variants of alternative genotype. In brief, 5′UTR amplicons were purified using the Nucleospin PCR Clean-up Kit (Macherey–Nagel) and quantified with the Qubit dsDNA HS Assay Kit (Thermo Fisher Scientific). Amplicon libraries were prepared according to Illumina standard protocols using indexing and sequenced on the Illumina MiSeq platform using 2× 300 bp sequencing kit (Illumina). The NGS data in FASTQ format were subjected to demultiplexing, adapter trimming and low-quality read removal using fastp (version 0.23.4). Sequence data quality control was then performed using FastQC (version 0.12.1). The summary quality control report was made using MultiQC (version 1.21). Read mapping to the reference (GenBank: AJ242654) was performed using BWA-MEM (version 0.7.17). SAM files were obtained, which were then converted to BAM files and sorted using samtools (version 1.15.1). Integrative Genomic Viewer version 2.19.1 was used to visualize the sequencing data.

### Assessment of exhaustion markers expression on CD4^+^ and CD8^+^ T-cells by flow cytometry

Pre-treatment programmed death receptor-1 (PD-1) and T cell immunoglobulin and mucin domain-containing protein 3 (Tim-3) expression on CD4^+^ and CD8^+^ T-cells were assessed as described previously^59^. In brief, isolated PBMC, resuspended in PBS were stained with BD Horizon Fixable Viability Stain 780 (BD Biosciences) and mixed with FcR blocking reagent (Miltenyi Biotec) following the manufacturer’s protocol. Next, one million cells were resuspended in Stain Buffer (BD Pharmingen), mixed with 5 µl of BV421 Mouse Anti-Human Tim-3 (CD366) Clone 7D3 (BD Horizon)), 5 µl of Alexa Fluor 647 Mouse Anti-Human PD-1 (CD279) Clone EH12.1, 5 µl of PerCP-Cy 5.5 Mouse Anti-Human CD3 Clone UCHT1, (both from BD Pharmingen), 5 µl of V500 Mouse Anti Human CD4 Clone RPA-TY (BD Horizon) and 1 µl of Mouse Anti-Human CD8 FITC Clone LT8 (ProImmune). Cells with added antibodies were incubated for 20 min at 4 °C, washed twice with PBS pH 7.2 (Life Technologies) and resuspended in 300 μL of Stain Buffer. The results were acquired immediately after staining by BD FACS Canto II Flow Cytometer (BD Biosciences), using BD FACS Diva version 6.0 program (BD Biosciences). Controls included unstained cells and isotype controls (Mouse Anti-Human IgG1 Alexa Fluor 647 and Mouse Anti-Human IgG1 BV421 instead of Alexa Fluor 647 Mouse Anti-Human PD-1 (CD279) and BV421 Mouse Anti-Human Tim-3 (CD366), respectively (both from BD Pharmingen).

For data analysis, the initial gate was set on lymphocytes on the forward scatter (FSC) *vs* side scatter (SSC) dot plot. Subsequently, singlet cells gate was set on FSC-H versus FSC-A dot plot. Next, based on SSC *vs* APC-Cy7 dot plot, only live cells were gated. Additionally, the following gates were employed: CD3^+^, CD4^+^, CD8^+^, PD-1^+^, Tim-3^+^ and PD-1^+^Tim-3^+^. The analysis was performed using BD FACS Diva version 6.0 program (BD Biosciences).

### Assessment of exhaustion markers plasma levels

IL-10, PD-1, Tim-3 and lymphocyte activation gene 3 (LAG-3) levels in plasma before and after treatment were assessed using Human IL-10 (High Sensitivity), PD-1, TIM-3, LAG-3 ELISA Kits (all from Thermo Fisher Scientific).

### Data and statistical analysis

Web based Basic Local Alignment Search Tool (BLAST) available at https://blast.ncbi.nlm.nih.gov/doc/blast was used to search for nucleotide similarity of the sequenced 5’UTR reads against the core nucleotide database, choosing option of Hepacivirus hominis (taxid:3052230) to determine the viral genotype. Additionally, a maximum likelihood phylogenetic analysis based on Tamura-Nei model of the sequenced region was conducted using MEGA version 11 against 1b, 3a, 4a, and 4d reference sequences (GenBank accession numbers: AJ242654, MN231293.1, DQ418782.1 and KP888621.1, respectively) to confirm evolutionary relationship of genotypes^60^.

Numerical data were graphically visualized using GraphPad program.

For the statistical analyses, Mann–Whitney, The Wilcoxon matched-pairs signed-ranks test and Fisher’s exact tests were used. Statistical analysis was performed using the GraphPad Prism program. All P-values were two-tailed and considered significant when < 0.05.

## Results

### HCV RNA may persist in PBMC after successful DAA treatment

Before treatment, HCV RNA in PBMCs was detectable in all (97, 100%) patients, whereas after treatment, in 9 (9.3%) patients (i.e., # 5, 6, 14, 36, 38, 47, 48, 52 and 82). Two patients (i.e., # 5 and 6) were treated with ombitasvir + paritaprevir + ritonavir + dasabuvir, six patients (i.e., # 14, 36, 38, 47, 48, and 52) were treated with ledipasvir + sofosbuvir, and one patient (i.e., # 82) was treated with elbasvir + grazoprevir (Table 2). Two patients with detectable virus experienced previous non-effective IFN-based treatment (Table 2). All these patients were diagnosed as infected with HCV 1b by routine INNO-LiPA HCV II testing of serum samples before treatment. In six out of nine HCV RNA-positive patients (i.e., #5, 6, 14, 47, 48 and 52), the 5’UTR pre-and post- treatment nucleotide sequence pairs derived from PBMC were obtained by Sanger-based sequencing (Table 2). The analysis revealed that the dominant PBMC sequences were distinct in patients # 5, 14, 47, 48 (i.e., differing by 4–18 point mutations), while in patient # 6 these were nearly identical (i.e., 2 point mutations), and in patient # 52—identical (i.e., no point mutations). The BLASTn and phylogenetic analysis showed that all pre-treatment PBMC sequences were 1b, concordant with the routine analysis in serum, whereas post-treatment PBMC sequences switched to sequences 3a in patient 5 and 48, to 4d in patient 14 and to 4a in patient 47, and remained 1b in patients 6 and 52 (Table 2 and Supplementary Fig. 1). NGS analysis revealed that in all patients in whom a genotype switch was observed, the respective pre-treatment PBMC and serum samples contained also a minor alternative genotype variants of low frequency carrying polymorphisms specific to this genotype (i.e., 0.01–0.75% and 0.11–0.94%, respectively), Table 2.

**Table 2:** Clinical and virological characteristics of patients in whom HCV RNA was detected in PBMC post-DAA treatment.

Patient number	DAA Treatment	History of previous CHC therapy (which)	Treatment duration [weeks]	Pre-treatment dominant HCV strain in serum	Pre-treatment dominant HCV genotype strain in PBMC	Age (years)	Sex [M/F]	BMI (kg/m^2^)	Liver fibrosis stage	ALT [IU/L]	HCV RNA [IU/mL]	Post-treatment dominant HCV genotype strain in PBMC	Minor alternative genotype strain detection in PBMC/serum before treatment (frequency %)
005	Ombitasvir + Paritaprevir + Ritonavir + Dasabuvir	No	12	1b	1b	38	F	42.1	F2	53	1,03 × 10^6^	3a	PBMC: 0.12–0.38%, serum: 0.11–0.26%
006	Ombitasvir + Paritaprevir + Ritonavir + Dasabuvir	No	12	1b	1b	40	M	37.7	F2	33	7,12 × 10^5^	1b	N/A
014	Ledipasvir + Sofosbuvir	Yes (IFN)	12	1b	1b	52	M	25.8	F2	115	2,37 × 10^5^	4d	PBMC: 0.12–0.75%, serum: 0.15–0.47%
036	Ledipasvir + Sofosbuvir	No	8	1b	N/A	81	F	25.0	F0/1	79	1,94 × 10^5^	N/A	N/A
038	Ledipasvir + Sofosbuvir	No	8	1b	N/A	73	F	29.6	F0/1	29	1,57 × 10^4^	N/A	N/A
047	Ledipasvir + Sofosbuvir	No	8	1b	1b	79	M	23.8	F0/1	34	5,01 × 10^5^	4a	PBMC: 0.01–0.18%, serum: 0.18–0.94%
048	Ledipasvir + Sofosbuvir	No	8	1b	1b	43	F	40.0	F0/1	53	1,30 × 10^5^	3a	PBMC: 0.11–0.17%, serum: 0.12–0.63%
052	Ledipasvir + Sofosbuvir	No	12	1b	1b	61	F	25.6	F0/1	255	6,17 × 10^3^	1b	N/A
082	Elbasvir + Grazoprevir	Yes (IFN)	12	1b	N/A	58	M	25.6	F0/1	63	1,28 × 10^4^	N/A	N/A

N/A, not available/not applicable

### Sex, liver fibrosis and previous IFN treatment are not associated with post-treatment OCI

We did not find the significant difference in the distribution of sex, degree of liver fibrosis, the type and the fact of previous IFN treatment between patients with OCI and no OCI (Supplementary Table 1).

### Patients with post-treatment OCI were characterized by a significantly lower pre-treatment viral load

**Fig. 1:**
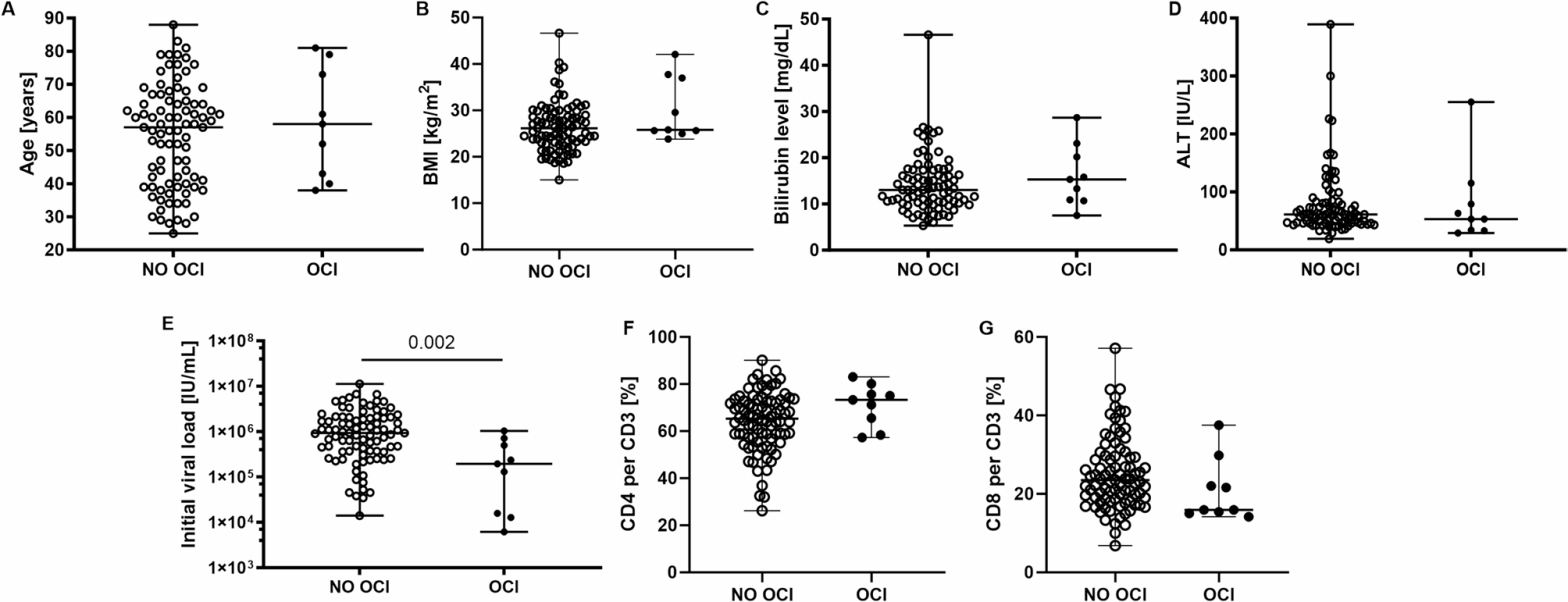
Baseline age (**A**), BMI (**B**), initial bilirubin levels (**C**), ALT activity (**D**), viral load (**E**), percentage of CD4^+^ (**F**) and CD8^+^ (**G**) T-cells in patients with undetectable (NO OCI) and detectable (OCI) HCV-RNA in PBMC after DAA treatment. Each point represents a single result, whiskers represent range, horizontal lines represent the median. *P*-value representing statistically significant difference in pairwise comparisons is indicated above the line.

Pre-treatment median age, BMI, bilirubin levels, ALT activity as well as percentage of CD4^+^ and CD8^+^ per CD3^+^ T-cells were not found to be significantly different in patients with OCI and no OCI (Fig. 1A–D,F,G, respectively and Supplementary Table 2). However, the median initial viral load was significantly lower in patients with OCI than in patients in whom no viral RNA was detected (1.9 × 10^5^ (6.2 × 10^3^–1.0 × 10^6^) vs 9.3 × 10^5^ (1.4 × 10^4^–1.1 × 10^7^) IU/mL, *P* = 0.002) (Fig. 1E).

### Patients with post-treatment OCI were characterized by a significantly lower pre-treatment Tim-3 expression on CD8^+^ T-cells

The median pre-treatment plasma IL-10, sPD-1**,** sTim-3 and sLAG-3 levels did not significantly differ between both groups of patients (Supplementary Table 2).

**Fig. 2:**
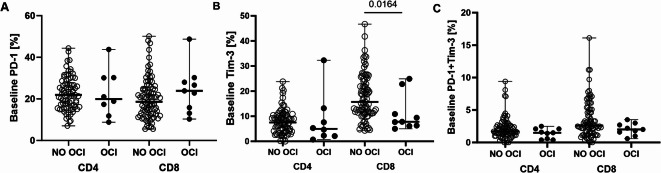
Flow cytometry analysis of percentages of CD4^+^ and CD8^+^ T-cells per total T-cells (i.e., CD3^+^) with membrane expression of PD-1 (**A**), Tim-3 (**B**) and PD-1 + Tim-3 (**C**) in patients with undetectable (NO OCI) and detectable (OCI) HCV-RNA in PBMC after DAA treatment. Each point represents a single result, whiskers represent range, horizontal lines represent the median. P-value representing statistically significant difference in pairwise comparisons is indicated above the line.

**Fig. 3:**
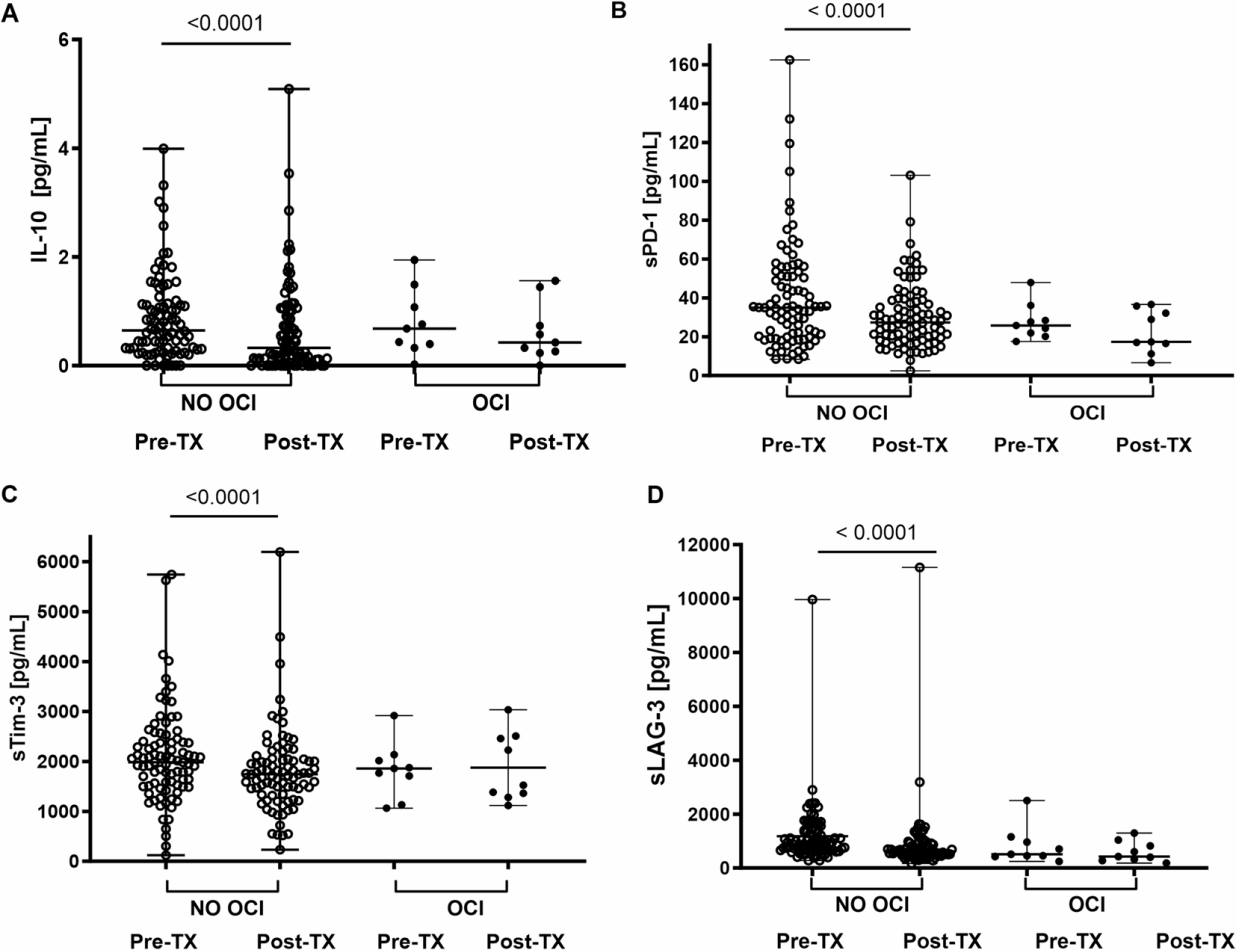
Baseline and post-treatment levels of IL-10 (**A**) sPD-1 (**B**), sTim-3 (**C**) and sLAG-3 (**D**) in plasma in patients with undetectable (NO OCI) and detectable (OCI) HCV-RNA in PBMC after DAA treatment. Each point represents a single result, whiskers represent range, horizontal lines represent the median. *P*-value representing statistically significant difference in pairwise comparisons is indicated above the line. Pre-TX, before treatment, Post-TX, post-treatment.

Although the median baseline percentages of PD-1 and PD-1 + Tim-3 expressing CD4^+^ and CD8^**+**^ T-cells did not significantly differ between the groups (Fig. 2A,C, respectively), we found that patients with OCI were characterized by significantly lower pre-treatment Tim-3 expression on CD8^+^ T-cells (7.8 (5.0–24.9) vs. 15.7 (4.3–46.7), *P* = 0.0164, Fig. 3B).

### Patients with post-treatment OCI did not experience a decrease in exhaustion markers levels

A significant decrease in plasma IL-10 level was observed in patients who eradicated the virus from PBMC (from 0.68 (0.02–1.94) to 0.32 (0.00–5.09) pg/mL, *P* < 0.0001), unlike in patients in whom the virus persisted (from 0.65 (0.00–3.99 to 0.42 (0.00–1.56) pg/mL, NS) (Fig. 3A). A similar pattern was observed for sPD-1 (a significant decrease in a former group from 35.0 (8.4–162.6) to 27.3 (2.4–103.2) pg/mL, *P* < 0.0001, no significant decrease in the latter group—from 25.8 (17.6–47.9) to 17.4 (6.6–36.7) pg/mL, NS) (Fig. 2B). The same was observed for sTim-3 (reduction from 1985.3 (120.8–5746.8) to 1744.9 (235.7–6195.1) pg/mL, P < 0.0001 vs from 1860.0 (1064.1–2919.3) to 1526.2 (1117.2–3035.1) pg/mL, NS, respectively) (Fig. 2C) and sLAG-3 (reduction from 898.3 (276.9–9962.0) to (615.8 (163.8–11,156.0) pg/mL, *P* < 0.0001 vs from 510.5 (244.3–2503.0) to 425.2 (181.1–1300.0) pg/mL, NS, respectively (Fig. 2D).

There were no significant differences in post-treatment IL-10, sPD-1**,** sTim-3 and sLAG-3 between non-OCI and OCI groups.

## Discussion

The primary aim of the presented study was to investigate the prevalence of OCI in patients after successful DAA treatment. We detected HCV-RNA in PBMC by ultrasensitive RT-PCR in all patients prior to treatment and in a relatively high percentage (i.e., 9.3%) of patients after the treatment. Other available DAA-based studies showed a similar scale of post-treatment OCI, ranging from 3.9 to 15%^16,31,61^. Interestingly, one of these studies reported the highest rate of OCI among patients after DAA treatment (15%) when compared to 10% after treatment with interferon and ribavirin, and 6.7% after spontaneous HCV clearance^16^.

To disclose the putative mechanism behind HCV persistence in PBMC, we sequenced the 5’UTR HCV-RNA fragment in patients in whom the virus was detected after treatment along with the pre-treatment samples. The analysis revealed that the dominant PBMC post-treatment sequences were distinct in majority (66.7%) of patients and included a genotype change from 1b to 3a or 4a/d, with concomitant detection of minor alternative genotype strain, both in pre-treatment serum and PBMC. These findings suggest that post-DAA OCI in these patients was a result of pre-existing mixed genotype infection and not a re-infection with the alternative genotype. The treatment in these patients was oriented to genotype 1 with adjusted doses, scheme and duration according to the recommendations and routine serum genotyping. Thus, it has most likely resulted in elimination of the dominant and susceptible genotype 1 strain and selection of alternative genotype strain to which the treatment could have been suboptimal. Indeed, the mixed infecting genotype may be unequally responsive to the same DAA drugs combination^62^.

Since the determinants of post-DAA OCI are poorly characterized, we analyzed the role of clinical parameters which could have predisposed to this condition, including age, sex, BMI, initial bilirubin concentration, ALT activity, viral load, liver fibrosis, the fact of previous HCV-oriented therapy and the therapeutic regimen. To reflect the possible immunological factors, we also investigated CD4/CD8 T-cell percentage as well as the levels of some immunoregulatory molecules expression on T-cells (PD-1 and Tim-3 iRs) and in plasma (IL-10, sPD-1, sLAG-3 and sTim-3). We found that patients with post-treatment OCI presented significantly lower initial median viral titer than the remaining subjects, indicating the predictive potential of baseline viral load testing. Our previous studies showed that lymphotropic variants displayed structural and genomic differences within an internal ribosome entry site (IRES) present within the 5’UTR, essential for translation of the viral polyprotein^63,64^. Such specialization of the lymphotropic quasispecies was at cost of a lower translational efficiency, possibly reflecting adaptation to the alternative cell types as a virus strategy of immune evasion^65,66^.

Interestingly, our study showed a distinct pre-treatment immunoregulatory profile (i.e., lower percentages of CD8^+^ T-cells expressing Tim-3) related to the occurrence of post-DAA OCI. Given the fact that Tim-3 expression characterizes terminally exhausted cells, in contrast to PD-1, which is related to T-cell activation and initial stages of exhaustion^67^, this suggests lower exhaustion. This could be due to observed in OCI patients lower viral load, and consequently, lower degree of antigenic stimulation known to be the driving force of exhaustion and Tim-3 expression^67^. Similar studies provided congruent findings on pre-existing immunoregulatory profile predisposing to HCV persistence after DAA, including the unfavorable (TT) IL28B genotype^19^ as well as significant elevation of neutrocyte-to-lymphocyte ratio, indicative of systemic inflammation^30^.

Importantly, patients who eradicated the virus from PBMC experienced a significant decrease in levels of immunoregulatory IL-10, sPD-1, sLAG-3, sTim-3, not observed in patients in whom the virus persisted. However, there were no significant differences in pre- and post-treatment levels of these markers between non-OCI and OCI groups. Thus, the level of significance may be more attributable to sample size than to actual biological differences and these results should be interpreted with caution as exploratory and rather hypothesis-generating.

The presented study did not demonstrate the role of the type of therapeutic regimen on the occurrence of OCI, which is congruent with a previous study^61^. Similarly, we also did not find the effect of other factors such as sex, age, BMI, fibrosis stage, bilirubin, ALT levels, or previous IFN-based treatment on the occurrence of post-treatment OCI. This again points to the importance of immuno-virological characteristics predisposing to this condition.

This study has limitations, since it does not involve further sequential follow-up to verify the dynamics of OCI. Unfortunately, patients are lost to follow-up from the Outpatient Clinic after SVR verification. Thus, we could not study the potential risk of future viral reactivation from PBMC reservoirs. Nevertheless, a relapse in OCI patients have been previously documented^16,22^. The lack of simultaneous HCV-RNA assessment in liver tissue may be also considered as a shortcoming, however, performing liver biopsy is ethically unjustified in such a study design and, as stated above, there is overall a good concordance between detection of HCV RNA between these two compartments^25^. While we detected post-DAA HCV sequences in PBMC, we could not assess whether they represent a replication-competent virus. Thus, the clinical significance of such findings requires cautious interpretation. Despite best efforts, due to the low HCV RNA titers in PBMCs, full-genome sequencing was technically unfeasible, and we could not provide these data. Thus, the genotype determination was based on 5’UTR analysis of which detection is conducted with the highest sensitivity. While it fails to correctly identify HCV subtypes 1a and 1b, it is variable enough for discrimination of HCV genotypes 1 to 5, which was sufficient for the purpose of the study^68^. Furthermore, we acknowledge that PCR may preferentially amplify certain viral variants and thus might have generated bias in their proportions, which could have an impact on our interpretation of data.

Finally, our findings rely on small number of observations of patients with post-treatment OCI, which may not reflect the real biological effect in vivo.

To conclude, our study showed a high rate of OCI (9.3%) in successfully DAA-treated patients according to the clinical criteria, which should instigate not only serum but also routine PBMC testing when assessing treatment outcome, as well as suggests the need of longer surveillance of these patients. In most cases, post-DAA OCI was related to switch of the dominant infecting genotype. Patients with post-treatment OCI displayed significantly lower pretreatment viral load and distinct pre-existing immune exhaustion status—a lower expression of Tim-3 on CD8^+^ T-cells.

## Electronic supplementary material

Below is the link to the electronic supplementary material.

Supplementary Material 1

Supplementary Material 2

Supplementary Material 3

## Data Availability

Pre- and post- treatment nucleotide sequence pairs derived from PBMC obtained by Sanger-based sequencing are available from Zenodo public repository ( 10.5281/zenodo.15038917). Next-generation sequencing data of pre-treatment serum and PBMC samples performed on Illumina Mi-Seq are available from Zenodo repository (10.5281/zenodo.15032240).
