# Supplementary material for: Persistence of hepatitis C virus in peripheral blood mononuclear cells of patients who achieved sustained virological response following treatment with direct-acting antivirals is associated with a distinct pre-existing immune exhaustion status
Source: Sci Rep. 2025 Jun 6;15:19918. doi: 10.1038/s41598-025-05084-z (PMC12144158; doi:10.1038/s41598-025-05084-z)
Supplement: Supplementary file 1 — Supplementary Material 1 [file 41598_2025_5084_MOESM1_ESM.docx]

*Supplementary Figure 1. Phylogenetic analysis of HCV 5’untranslated region in PBMC of patients before and after (F-marked) successful DAA treatment. The evolutionary history was inferred by using the Maximum Likelihood method and Tamura-Nei model against 1b, 3a, 4a, and 4d reference sequences (GenBank accession numbers: AJ242654, MN231293.1, DQ418782.1 and KP888621.1, respectively).*
