# Supplementary material for: Persistence of hepatitis C virus in peripheral blood mononuclear cells of patients who achieved sustained virological response following treatment with direct-acting antivirals is associated with a distinct pre-existing immune exhaustion status
Source: Sci Rep. 2025 Jun 6;15:19918. doi: 10.1038/s41598-025-05084-z (PMC12144158; doi:10.1038/s41598-025-05084-z)
Supplement: Supplementary file 2 — Supplementary Material 2 [file 41598_2025_5084_MOESM2_ESM.docx]

*Supplementary Table 1. Distribution of sex, degree of liver fibrosis and previous DAA treatment in groups of patients in whom HCV RNA was detected (OCI) and not detected (NO OCI) in PBMC after successful DAA treatment*

|  | Post- treatment OCI  n=9 | No OCI  n=88 | P-value |
| --- | --- | --- | --- |
| Sex [M/F] 4/5 | | 32/56 | 0.723 |
| Advanced liver fibrosis (F2/F3) | 3 (33%) | 38 (43%) | 0.729 |
| Treatment scheme  Harvoni (Ledipasvir+Sofosbuvir)  Viekirax+Exviera (Ombitasvir+Paritaprevir+ Ritonavir+Dasabuvir)  Zepatier (Elbasvir+Grazoprevir) | 6 (67%)  2 (22%)  1 (11%) | 63 (72%)  19 (22%)  6 (7%) | 0.715  0.715  1.000 |
| Previous unsuccessful IFN-based treatment history | 2 (22%) | 21 (24.0%) | 1.000 |
|  |  |  |  |
