# Supplementary material for: Persistence of hepatitis C virus in peripheral blood mononuclear cells of patients who achieved sustained virological response following treatment with direct-acting antivirals is associated with a distinct pre-existing immune exhaustion status
Source: Sci Rep. 2025 Jun 6;15:19918. doi: 10.1038/s41598-025-05084-z (PMC12144158; doi:10.1038/s41598-025-05084-z)
Supplement: Supplementary file 3 — Supplementary Material 3 [file 41598_2025_5084_MOESM3_ESM.docx]

*Supplementary Table 2. Pre-treatment clinical and immunological characteristics of patients in whom HCV RNA was detected (OCI) and not detected (NO OCI) in PBMC after successful DAA treatment*

|  | Post- treatment OCI  n=9 | No OCI  n=88 | P-value |
| --- | --- | --- | --- |
| Age [years, median (range)]. 58 (38-81)  BMI [kg/m^2^] 25.8 (23.8-42.1) | | 57 (25-88)  26.1 (15.0-46.7) | 0.5260  0.1309 |
|  |  |  |  |
| Initial ALT [median (range)] | 53 (19-389) | 61 (29-255) | 0.5886 |
| Initial viral load [IU/mL, median (range)] | 1.9×10^5^ (6.2×10^3^-1.0×10^6^) | 9.3×10^5^ (1.4×10^4^-1.1×10^7^) | 0.002 |
| Initial bilirubin [mg/dL, median (range)] | 15.3 (7.5-28.7) | 13.0 (5.3-46.6) | 0.3260 |
| CD4 per CD3^+^ cells [%] | 73.3 (57.3-83.00) | 65.3 (26.2-90.1) | 0.0959 |
| CD8 per CD3^+^ cells [%]  IL-10 [pg/mL, median (range)]  sPD-1 [pg/mL, median (range)]  sTim-3[pg/mL, median (range)]  sLAG-3 [pg/mL, median (range)] | 15.9 (14.2-37.5) | 23.5 (14.2-37.5) | 0.0934 |
|  | 0.68 (0.022-1.944)  25.8 (17.6-47.9)  1860.0 (1064.1-2919.3)  510.5 (244.3-2503.0) | 0.647 (0.000-3.992)  35.0 (8.4-162.6)  1985.3 (120.8-5746.8)  898.3 (276.9-9962.0) | 0.9702  0.2088  0.3790  0.0561 |
